# A revised model for the role of GacS/GacA in regulating type III secretion by *Pseudomonas syringae* pv. *tomato* DC3000

**DOI:** 10.1111/mpp.12876

**Published:** 2019-10-07

**Authors:** Megan R. O’Malley, Ching‐Fang Chien, Scott C. Peck, Nai‐Chun Lin, Jeffrey C. Anderson

**Affiliations:** ^1^ Department of Botany and Plant Pathology Oregon State University Corvallis OR USA; ^2^ Department of Agricultural Chemistry National Taiwan University Taipei Taiwan R.O.C.; ^3^ Department of Biochemistry University of Missouri Columbia MO USA; ^4^ Christopher S Bond Life Sciences Center University of Missouri Columbia MO USA; ^5^ Interdisciplinary Plant Group University of Missouri Columbia MO USA; ^6^ Institute of Plant and Microbial Biology Academia Sinica Taipei Taiwan R.O.C.

**Keywords:** GacS/GacA, plant–microbe interactions, *Pseudomonas syringae*, two‐component regulatory systems, type III secretion, virulence regulation

## Abstract

GacS/GacA is a conserved two‐component system that functions as a master regulator of virulence‐associated traits in many bacterial pathogens, including *Pseudomonas* spp., that collectively infect both plant and animal hosts. Among many GacS/GacA‐regulated traits, type III secretion of effector proteins into host cells plays a critical role in bacterial virulence. In the opportunistic plant and animal pathogen *Pseudomonas aeruginosa*, GacS/GacA negatively regulates the expression of type III secretion system (T3SS)‐encoding genes. However, in the plant pathogenic bacterium *Pseudomonas syringae*, strain‐to‐strain variation exists in the requirement of GacS/GacA for T3SS deployment, and this variability has limited the development of predictive models of how GacS/GacA functions in this species. In this work we re‐evaluated the function of GacA in *P. syringae* pv. *tomato* DC3000. Contrary to previous reports, we discovered that GacA negatively regulates the expression of T3SS genes in DC3000, and that GacA is not required for DC3000 virulence inside *Arabidopsis* leaf tissue. However, our results show that GacA is required for full virulence of leaf surface‐inoculated bacteria. These data significantly revise current understanding of GacS/GacA in regulating *P. syringae* virulence.

Many bacterial pathogens can survive outside of their hosts yet must transition into more virulent forms during initial stages of infection. *Pseudomonas syringae* is a Gram‐negative plant pathogenic bacterium that infects the interior of leaf tissue by swimming through natural openings or wounds in the leaf surface (Abramovitch *et al.*, 2006; Xin *et al.*, 2018). Once inside, *P syringae* switches from motile to sessile and begins secreting immunity‐suppressing effectors into host cells via a type III secretion system (T3SS), a lifestyle switch necessary for *P. syringae* to cause disease (Abramovitch *et al.*, 2006; Schreiber and Desveaux, 2011; Xin *et al.*, 2018). How motility and T3SS deployment, as well as other virulence‐associated traits, are coordinately regulated in *P. syringae* to mediate this lifestyle transition is poorly understood.

GacS/GacA is a highly conserved two‐component system in γ‐proteobacteria, and in many pathogenic species is a master regulator of virulence‐associated traits (Heeb and Haas, 2001). A role for GacS/GacA in regulating bacterial virulence was first established through studies of *P. syringae* over 25 years ago (Willis *et al.*, 1990), and GacS/GacA homologues have since been shown to regulate the virulence of many pathogens, including *Vibrio cholera* (Wong *et al.*, 1998), *Salmonella typhimurium* (Johnston *et al.*, 1996), and the opportunistic plant and animal pathogen *Pseudomonas aeruginosa* (Heeb and Haas, 2001; Rahme *et al.*, 2019). In *P.aeruginosa*, GacS/GacA negatively regulates T3SS deployment and motility, and positively regulates biofilm formation and type VI secretion, among other factors (Valentini *et al.*, 2018). In *P. syringae*, GacS/GacA functions as a master regulator of multiple virulence traits, including T3SS deployment, toxin production and motility (Chatterjee *et al.*, 2003; Heeb and Haas, 2001; Mole *et al.*, 2007). However, significant strain‐to‐strain differences in phenotypes of *gacS* ^−^ and *gacA* ^−^ mutants have been reported. For instance, a *gacS* ^−^ mutant of the bean pathogen *P. syringae* pv. *syringae* B728a had decreased field fitness yet had sufficient levels of type III secretion to trigger a host defence response and was fully virulent in laboratory infections of host plants (Hirano *et al.*, 1997; Willis *et al.*, 1990). In contrast, a *gacA* ^−^ mutant of *P. syringae* pv. *syringae* DC3000, a pathogen of *Arabidopsis* and tomato, had decreased T3SS gene expression and was less virulent on host plants (Chatterjee *et al.*, 2003; Vargas *et al.*, 2013). Because DC3000 is one of the most intensively studied plant pathogens, these results largely established GacS/GacA as a positive regulator of T3SS deployment in *P. syringae* (Brencic and Winans, 2005; Mole *et al.*, 2007; Tang *et al*., 2006). However, the apparently conflicting modes of GacS/GacA‐T3SS regulation between DC3000, B728a (Hirano *et al.*, 1997; Willis *et al.*, 1990; Yu *et al.*, 2014) and other strains (Marutani *et al.*, 2008) suggest that GacS/GacA functions have diversified at the level of individual *P. syringae* isolates, and this variability has complicated efforts to establish predictive species‐level models of how GacS/GacA regulates *P. syringae* virulence.

Here we re‐examined the virulence‐associated phenotypes of strain AC811, a DC3000 Tn*5*::*gacA* mutant (Chatterjee *et al.*, 2003; Ferreiro *et al.*, 2018; Vargas *et al.*, 2013). Contrary to previous reports (Chatterjee *et al.*, 2003; Vargas *et al.*, 2013), we demonstrate that AC811 hyper‐expresses T3SS‐encoding genes in culture and during plant infection. We further show that GacA is dispensable for DC3000 virulence in the leaf interior but is required for virulence of leaf surface‐inoculated bacteria, most likely due to motility defects caused by loss of *gacA*. Together, these results significantly revise current understanding of how GacS/GacA functions to regulate type III secretion by *P. syringae* (Brencic and Winans, 2005; Lapouge *et al.*, 2008; MacLean and Studholme, 2010; Mole *et al.*, 2007; Tang *et al*., 2006). These data provide a solid framework for modelling and testing future hypotheses of how GacS/GacA regulates lifestyle switching of *P. syringae* during plant infection.

We initially assessed whether GacA is required for DC3000 to respond to plant‐derived metabolic signals that induce the expression of T3SS‐encoding genes in DC3000 (Anderson *et al.*, 2014). To investigate, we introduced a transcriptional reporter consisting of the promoter of T3SS effector gene *avrPto* fused to *green fluorescent protein* (*gfp*) into DC3000 and AC811. We then cultured these reporter strains in minimal medium supplemented with T3SS‐inducing fructose and citric acid (Anderson *et al.*, 2014; Huynh *et al.*, 1989). AC811 expressed higher levels of *avrPto* in response to the bioactive metabolites (Fig. 1A). Increased *avrPto* expression occurred in metabolite‐treated AC811 cultures inoculated from King’s B (KB) agar (Fig. 1A) or KB broth cultures (Fig. S1). We confirmed these results by immunoblot detection of AvrPto produced by expression of endogenous *avrPto* (Fig. 1B) and by qRT‐PCR of *avrPto* mRNA levels (Fig. S2A). Over‐expression of *gacA* in AC811 fully restored *avrPto* expression back to DC3000 levels (Fig. S3). We also used allelic exchange to delete *gacA* in DC3000 (Fig. S4) and measured similar heightened *avrPto* expression in the resulting Δ*gacA*‐1 mutant strain (Fig. 1C), as well as significantly increased transcript levels of the T3SS master regulator *hrpL* (Fig. S2A).

**Figure 1:**
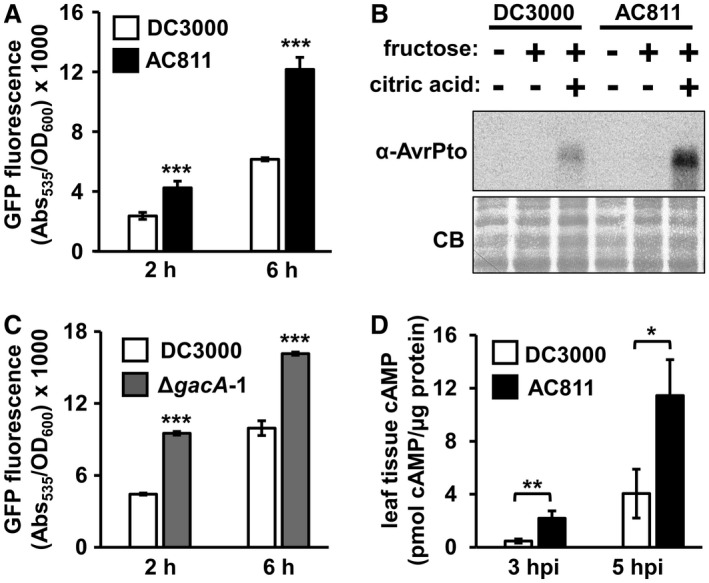
GacA negatively regulates type III secretion in *Pseudomonas syringae* pv. *tomato* DC3000. (A) GFP fluorescence of DC3000 and AC811 *avrPto_promoter_*:*gfp* reporter strains. Bacteria were incubated in minimal medium (MM) with 10 mM fructose and 400 µM citric acid. Graphed are means ± SE of GFP fluorescence normalized to OD_600_ and background fluorescence from empty vector strains, *n* = 4. Data are representative of three independent experiments. Asterisks denote significant difference between strains based on *t*‐test, ***P < 0.001. (B) AvrPto levels in DC3000 and AC811 incubated in MM supplemented with 200 µM citric acid and/or 10 mM fructose as indicated. Upper panel is immunoblot detection of AvrPto in treated bacteria after 5 h. Lower panel is Coomassie Brilliant Blue (CB) staining of blot as a loading control. (C) GFP fluorescence of DC3000 and Δ*gacA*‐1 carrying *avrPto*_promoter_:*gfp* reporter plasmids. Bacteria were incubated in MM with 10 mM fructose and 400 µM citric acid. Graphed are means ± SE of GFP fluorescence normalized to OD_600_ and background fluorescence from empty vector strains, *n* = 4. Data are representative of three independent experiments. Asterisks denote significant difference between strains based on *t*‐test, ***P < 0.001. (D) cAMP levels in *Arabidopsis* leaves infected with DC3000 or AC811 strains carrying an AvrPto‐adenylate cyclase reporter (AvrPto‐CyaA). Graphed data are means ± SE of cAMP levels in infected tissue sampled at 3 and 5 h post‐infection (hpi), normalized to total protein content of samples. Data were pooled from three independent experiments, *n* = 9. **P < 0.01; *P < 0.05 based on *t*‐test.

In light of these results, we next sought to determine whether AC811 similarly hyper‐expresses its T3SS *in planta.* The hypersensitive response (HR) is a localized cell death phenotype caused by immune receptor‐mediated recognition of pathogen effectors inside plant cells (Dangl and Jones, 2001). *Pseudomonas syringae* mutants that cannot deliver effectors are unable to trigger an HR. In this regard, AC811 was reported to elicit a weaker HR in tobacco leaves (Chatterjee *et al.*, 2003). We re‐examined this phenotype in tobacco using an ion leakage assay and observed that T3SS‐dependent HR cell death induced by AC811 and Δ*gacA*‐1 was at least equivalent to DC3000 (Fig. S5). To measure effector delivery in a more quantitative manner, we used an adenylate cyclase (CyaA) reporter assay to quantify the amount of AvrPto‐CyaA delivered by AC811 into plant cells during infection of *Arabidopsis* (Miao *et al.*, 1999; Schechter *et al.*, 2004). Consistent with the increased *avrPto* expression in cultured AC811, we measured significantly higher levels of AvrPto‐CyaA delivery in AC811‐infected leaf tissue (Fig. 1D). We conclude from these data that GacA negatively regulates T3SS deployment by DC3000 when cultured with defined T3SS‐inducing metabolite signals and during plant infection.

Increased expression and delivery of type III effectors by AC811 was unexpected based on the reported virulence defect of this strain. To re‐evaluate the role of GacA in regulating DC3000 virulence, we infiltrated DC3000, AC811 and Δ*gacA*‐1 into the interior, or apoplast, of *Arabidopsis* leaves. Three days post‐infection we observed a significant reduction in visible disease symptoms caused by AC811, as previously reported (Fig. 2A) (Chatterjee *et al.*, 2003). We also measured a significant decrease in the number of bacteria in AC811‐infected tissue (Fig. 2B). However, Δ*gacA*‐1 did not phenocopy AC811 and instead caused disease symptoms and grew to levels comparable to those of DC3000 (Fig. 2A,B). To confirm that these conflicting results were not due to variation in laboratory strains of DC3000 (Landgraf *et al.*, 2006), we deleted *gacA* in DC3000 isolates obtained from two other laboratories (Table S1) and observed no significant decrease in the virulence of these mutants (Fig. S6). Also, *gacA* over‐expression did not rescue the virulence defect of AC811 (Fig. 2C). Based on these results, we conclude that GacA is not required for DC3000 virulence within *Arabidopsis* leaf tissue, and that loss of GacA is not responsible for the virulence defect of AC811. In follow‐up experiments we discovered that a polar effect of Tn*5*::*gacA* on downstream *uvrC* expression and a nonsense mutation in cell wall recycling enzyme *anmK* are responsible for decreased virulence of AC811 (O'Malley *et al*., 2019).

**Figure 2:**
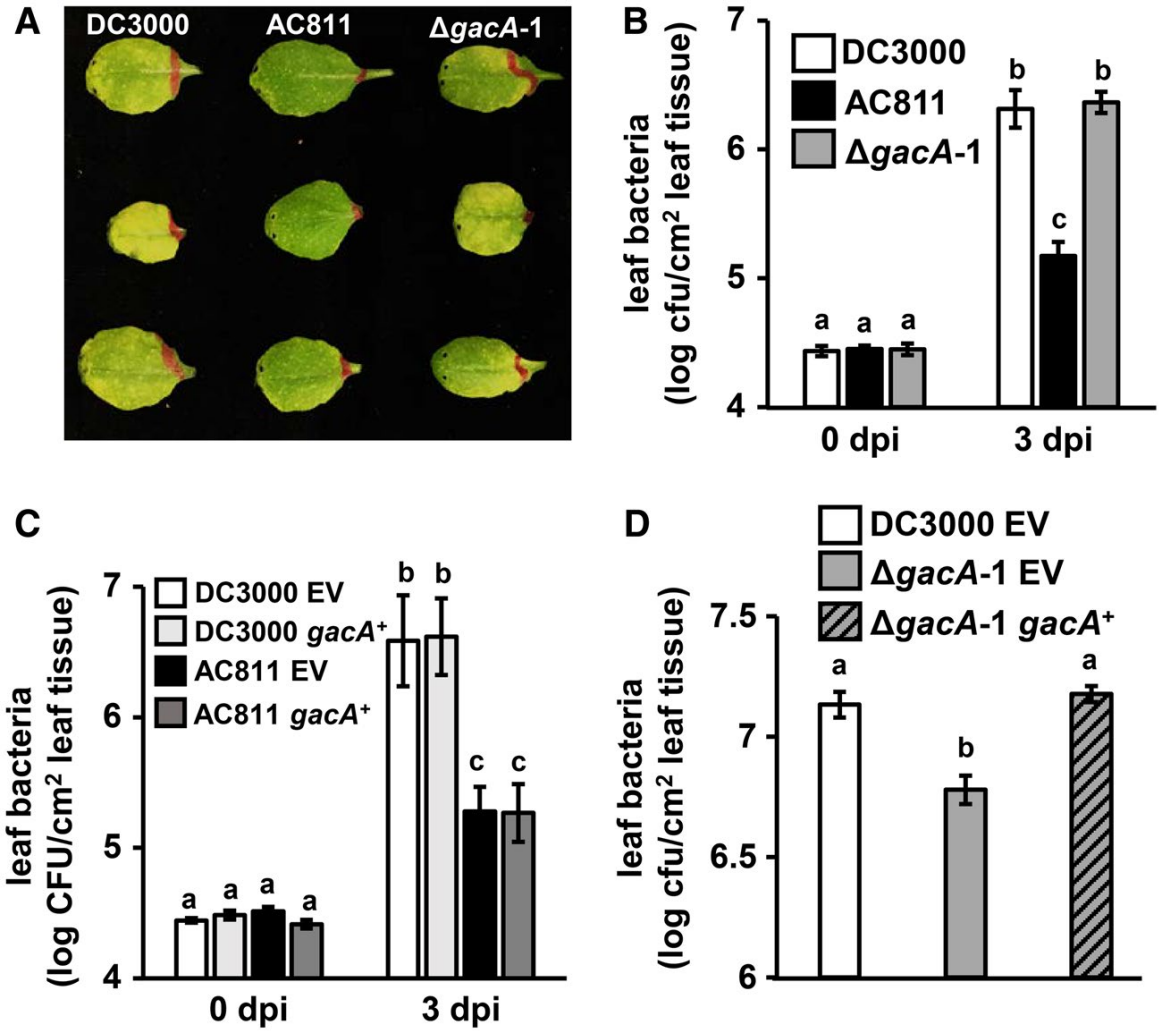
GacA is not required for virulence of DC3000 within the apoplast but is required for growth of leaf‐surface inoculated DC3000. (A) Leaves of 4‐week‐old *Arabidopsis* plants were syringe‐infiltrated with DC3000, AC811 or Δ*gacA*‐1. Shown is a photograph of disease symptoms on detached leaves 3 days post‐infection (dpi). (B) Growth of DC3000, AC811 and Δ*gacA*‐1 in leaves of 4‐week‐old plants infected by syringe‐infiltration. Graphed are means ± SE of colony‐forming units (cfus) in leaves based on serial dilution plating, *n* = 9. Data were pooled from three independent experiments. (C) DC3000 or AC811 carrying plasmids with *gacA* under native promoter control (*gacA* ^+^) were syringe‐infiltrated into *Arabidopsis* Col‐0 leaves and bacterial levels measured by serial dilution plating. EV, empty vector. Graphed are means ± SE of cfus in leaves based on serial dilution plating, *n* = 3. Data are representative of three independent experiments. (D) Bacterial populations in 5‐week‐old leaves infection by spray‐inoculation. Graphed are means ± SE of bacterial levels 3 dpi based on serial dilution plating, *n* = 6. Data are representative of three independent experiments. Lower case letters in panels B to D denote statistical groups determined by ANOVA with multiple pairwise *t*‐test comparisons and Tukey’s post hoc HSD analysis, *P* < 0.05.

In addition to regulation of T3SS, GacS/GacA was previously reported to positively regulate DC3000 motility based on swimming and swarming defects of AC811 (Chatterjee *et al.*, 2003; Ferreiro *et al.*, 2018; Vargas *et al.*, 2013). We tested both AC811 and our Δ*gacA*‐1 strain on swim agar plates and confirmed that GacA is required for swimming motility (Fig. S7). Because motility is necessary for invasion of leaf tissue by *P. syringae*, we reasoned that Δ*gacA*‐1 may be less virulent when inoculated onto a leaf surface. To investigate, we sprayed *Arabidopsis* leaves with suspensions of DC3000, Δ*gacA*‐1 or a *gacA*‐complemented Δ*gacA*‐1 strain. In contrast to apoplast infection, we measured a significant decrease in the population of bacteria on Δ*gacA*‐1‐infected leaves, and this phenotype was fully complemented by expression of *gacA* (Fig. 2D). We conclude that GacA is required for full virulence of leaf surface‐inoculated DC3000.

In summary, our results indicate that GacA functions as a negative regulator of type III secretion in DC3000. Our data also demonstrate that GacA is not required for virulence of DC3000 in the leaf apoplast, but is required for full virulence of DC3000 on the leaf surface, most likely due to its role in regulating motility. We propose a model in which GacS/GacA regulates a lifestyle switch of *P. syringae* by inversely regulating motility and T3SS during host infection. In this model, GacS/GacA is activated on the leaf surface, thereby promoting motility and dampening T3SS deployment, and deactivated during apoplast colonization, allowing for decreased motility and increased type III secretion to suppress host immunity. GacS/GacA may coordinately regulate additional virulence‐associated traits such as toxin production and biofilm formation in a similar manner. Testing this model will require closer examination of when, where and how GacS/GacA is activated during leaf infection, as well as examining signalling pathways downstream of GacS/GacA activation and how they may coordinately regulate both motility and T3SS.

## Supporting information


**Fig. S1** Hyper‐expression of *avrPto* occurs in AC811 cultured in KB broth prior to fructose and citric acid treatment. GFP fluorescence of DC3000 and AC811 strains carrying an *avrPto_promoter_*:*gfp* reporter plasmid. Bacteria were first cultured overnight in KB broth then incubated in a minimal medium (MM) with 10 mM fructose and 400 µM citric acid. Graphed are means ± SE of GFP fluorescence at 6 h post‐inoculation normalized to OD_600_ and fluorescence from pProbe‐GT empty vector strains, *n* = 4. Asterisks denote significant difference based on *t*‐test, *P* < 0.001. Data are representative of three independent experiments.

**Fig. S2** GacA negatively regulates the abundance of mRNA transcripts from T3SS‐associated genes. DC3000, AC811 and Δ*gacA*‐1 were incubated in minimal medium (MM) with 10 mM fructose and 400 µM citric acid for 2 h. The abundance of *avrPto *and *hrpL *transcripts in treated bacteria was measured by quantitative RT‐PCR. Transcript abundance was normalized to (A) *gyrA* or (B) *ffh* reference genes, followed by normalization to transcript levels in DC3000. Graphed are means ± SE from data pooled from three independent experiments, *n* = 10. Asterisks denote statistical significance as determined by pairwise *t*‐tests between DC3000 and indicated mutant strains. *P < 0.05; ***P < 0.001; n.s., no significant difference. (C) Threshold cycle (Ct) values from quantitative RT‐PCR of reference genes *gyrA* (left) and *ffh* (right). Graphed are means ± SE from data pooled from three independent experiments, *n* = 10. Statistical significance was determined by pairwise *t*‐tests; n.s., no significant difference based on *P* > 0.05.

**Fig. S3** Expression of *gacA *complements the *avrPto* hyper‐expression phenotype of AC811. (A) Abundance of *gacA* transcripts measured by qRT‐PCR using *gacA*‐specific primers*. gacA *transcripts were normalized to transcripts from *gyrA*. Graphed are means ± SE, with data pooled from two independent experiments, *n* = 8. ***P < 0.001 based on two sample *t*‐test comparisons with DC3000. (B) GFP fluorescence of DC3000 and AC811 *avrPto_promoter_*:*gfp* reporter strains carrying either a *gacA*‐complementing plasmid (*gacA* ^+^) or empty vector (EV). Bacteria were incubated in minimal medium (MM) with 10 mM fructose and 400 µM aspartic acid. Graphed are means ± SE of GFP fluorescence at 12 h post‐inoculation normalized to OD_600_ and fluorescence from pProbe‐GT empty vector strains, *n* = 9. Data are pooled from three independent experiments. Asterisks denote significant difference between strains based on *t*‐test, *P* < 0.001.

**Fig. S4** PCR genotyping of Δ*gacA*‐1 confirms deletion of *gacA*. A fragment of DNA containing the *gacA *open reading frame was PCR‐amplified from DC3000 or Δ*gacA*‐1 genomic DNA. Shown are PCR products separated by agarose gel electrophoresis and visualized by ethidium bromide staining.

**Fig. S5** Loss of *gacA* does not decrease the hypersensitive response in non‐host tobacco leaves. Leaves of *Nicotiana tabacum *cultivar KY21 were syringe‐infiltrated with DC3000 and DC3000‐derived mutants, including a T3SS‐deficient *hrcC* ^‐^ strain. (A) Photograph of a leaf 8 h post‐infiltration with 1 × 10^8^ cfu/mL (left) or 1 × 10^7^ cfu/mL (right) of bacteria. Labels are 1, 2, Δ*hrcC*; 3, 4, DC3000; 5, 6, AC811; 7, 8, Δ*gacA‐1*. Image is representative of three independent experiments. (B) Leaf disks (five disks/infected area) were taken at 8 h post‐infiltration and incubated in 5 mL of H_2_O for 1 h. Ion leakage from leaf tissue was quantified by conductivity meter. Graphed are means ± SE of conductivity measurements, *n* = 9. Data are pooled from three independent experiments. Lower case letters denote statistical groups determined by ANOVA with multiple pairwise *t*‐test comparisons and Tukey’s post hoc HSD analysis,* P* < 0.05.

**Fig. S6** GacA is not required for virulence of DC3000 syringe‐infiltrated into *Arabidopsis* leaves. Growth of Δ*gacA *deletion mutants in *Arabidopsis *leaves infected by syringe infiltration. Solid colours indicate DC3000 obtained from G. Martin (Cornell) and its corresponding mutant Δ*gacA‐*2; dotted bars indicate DC3000 obtained from B. Kunkel (Wash U) and its corresponding mutant Δ*gacA‐*3. Graphed are means ± SE of colony‐forming units (cfus) in leaves based on serial dilution plating of leaf tissue extracts, *n* = 6. Data were pooled from two independent experiments. dpi, days post‐infection; ns, not significant based on ANOVA with multiple pairwise *t*‐test comparisons and Tukey’s post hoc HSD analysis,* P* < 0.05.

**Fig. S7** GacA positively regulates motility of DC3000. DC3000, AC811 and *ΔgacA*‐1 were individually spotted onto King's B medium (KBM) agar plates containing 0.25% agar to detect swimming motility. (A) Photographs of bacteria on swimming motility plates after 24 h. White bars show scale of 1 cm. (B) Graphed are means ± SE of radii of bacterial spread measured after 24 h on swim plates, *n* = 4. Data are representative of three independent experiments.

**Table S1** Sequences of oligonucleotide primers used in this study.

**Table S2** List of bacterial strains used in this study.

**Methods S1** Experimental Procedures.

## Data Availability

The data that support the findings of this study are available from the corresponding author upon request.

## References

[mpp12876-bib-0001] Abramovitch, R.B. , Anderson, J.C. and Martin, G.B. (2006) Bacterial elicitation and evasion of plant innate immunity. Nat. Rev. Mol. Cell Biol. 7, 601–611. 16936700 10.1038/nrm1984 PMC2842591

[mpp12876-bib-0002] Anderson, J.C. , Wan, Y. , Kim, Y.‐M. , Pasa‐Tolic, L. , Metz, T.O. and Peck, S.C. (2014) Decreased abundance of type III secretion system‐inducing signals in *Arabidopsis mkp1* enhances resistance against *Pseudomonas syringae* . Proc. Natl. Acad. Sci. USA, 111, 6846–6851. 24753604 10.1073/pnas.1403248111 PMC4020108

[mpp12876-bib-0003] Brencic, A. and Winans, S.C. (2005) Detection of and response to signals involved in host–microbe interactions by plant‐associated bacteria. Microbiol. Mol. Biol. Rev. 69, 155–194. 15755957 10.1128/MMBR.69.1.155-194.2005 PMC1082791

[mpp12876-bib-0004] Chatterjee, A. , Cui, Y. , Yang, H. , Collmer, A. , Alfano, J.R. and Chatterjee, A.K. (2003) GacA, the response regulator of a two‐component system, acts as a master regulator in *Pseudomonas syringae* pv. *tomato* DC3000 by controlling regulatory RNA, transcriptional activators, and alternate sigma factors. Mol. Plant–Microbe Interact. 16, 1106–1117. 14651344 10.1094/MPMI.2003.16.12.1106

[mpp12876-bib-0005] Dangl, J.L. and Jones, J.D.G. (2001) Plant pathogens and integrated defense responses to infection. Nature, 411, 826–833. 11459065 10.1038/35081161

[mpp12876-bib-0006] Ferreiro, M.‐D. , Nogales, J. , Farias, G.A. , Olmedilla, A. , Sanjuán, J. and Gallegos, M.T. (2018) Multiple CsrA proteins control key virulence traits in *Pseudomonas syringae* pv. *tomato* DC3000. Mol. Plant–Microbe Interact. 31, 525–536. 29261011 10.1094/MPMI-09-17-0232-R

[mpp12876-bib-0007] Heeb, S. and Haas, D. (2001) Regulatory roles of the GacS/GacA two‐component system in plant‐associated and other Gram‐negative bacteria. Mol. Plant–Microbe Interact. 14, 1351–1363. 11768529 10.1094/MPMI.2001.14.12.1351

[mpp12876-bib-0008] Hirano, S.S. , Ostertag, E.M. , Savage, S.A. , Baker, L.S. , Willis, D.K. and Upper, C.D. (1997) Contribution of the regulatory gene *lemA* to field fitness of *Pseudomonas syringae* pv. *syringae* . Appl. Environ. Microbiol. 63, 4304–4312. 16535727 10.1128/aem.63.11.4304-4312.1997 PMC1389283

[mpp12876-bib-0009] Huynh, T.V. , Dahlbeck, D. and Staskawicz, B.J. (1989) Bacterial blight of soybean: regulation of a pathogen gene determining host cultivar specificity. Science, 245, 1374–1377. 2781284 10.1126/science.2781284

[mpp12876-bib-0010] Johnston, C. , Pegues, D.A. , Christoph, J. , Lee, C.A. and Miller, S.I. (1996) Transcriptional activation of *Salmonella typhimurium* invasion genes by a member of the phosphorylated response‐regulator superfamily. Mol. Microbiol. 22, 715–727. 8951818 10.1046/j.1365-2958.1996.d01-1719.x

[mpp12876-bib-0011] Landgraf, A. , Weingart, H. , Tsiamis, G. and Boch, J. (2006) Different versions of *Pseudomonas syringae* pv. *tomato* DC3000 exist due to the activity of an effector transposon. Mol. Plant Pathol. 7, 355–364. 20507452 10.1111/j.1364-3703.2006.00343.x

[mpp12876-bib-0012] Lapouge, K. , Schubert, M. , Allain, F.H.T. and Haas, D. (2008) Gac/Rsm signal transduction pathway of γ‐proteobacteria: From RNA recognition to regulation of social behaviour. Mol. Microbiol. 67, 241–253. 18047567 10.1111/j.1365-2958.2007.06042.x

[mpp12876-bib-0013] MacLean, D. and Studholme, D.J. (2010) A Boolean model of the *Pseudomonas syringae hrp* regulon predicts a tightly regulated system. PLoS ONE, 5, E9101. 20169167 10.1371/journal.pone.0009101 PMC2821412

[mpp12876-bib-0014] Marutani, M. , Taguchi, F. , Ogawa, Y. , Hossain, M.M. , Inagaki, Y. , Toyoda, K. , Shiraishi, T. and Ichinose, Y. (2008) Gac two‐component system in *Pseudomonas syringae* pv. *tabaci* is required for virulence but not for hypersensitive reaction. Mol. Genet. Genomics, 279, 313–322. 18080141 10.1007/s00438-007-0309-y

[mpp12876-bib-0015] Miao, E.A. , Scherer, C.A. , Tsolis, R.M. , Kingsley, R.A. , Adams, L.G. , Bäumler, A.J. and Miller, S.I. (1999) *Salmonella typhimurium* leucine‐rich repeat proteins are targeted to the SPl1 and SPl2 type III secretion systems. Mol. Microbiol. 34, 850–864. 10564523 10.1046/j.1365-2958.1999.01651.x

[mpp12876-bib-0016] Mole, B.M. , Baltrus, D.A. , Dangl, J.L. and Grant, S.R. (2007) Global virulence regulation networks in phytopathogenic bacteria. Trends Microbiol. 15, 363–371. 17627825 10.1016/j.tim.2007.06.005

[mpp12876-bib-0017] O'Malley, M.R. , Weisberg, A.J. , Chang, J.H. and Anderson, J.C. (2019) Re‐evaluation of a Tn*5*::*gacA* mutant of *Pseudomonas syringae* pv. *tomato* DC3000 uncovers roles for *uvrC* and *anmK* in promoting virulence. BioRxiv. 10.1101/774711. PMC6786584 31600319

[mpp12876-bib-0018] Rahme, L.G. , Stevens, E.J. , Wolfort, S.F. , Shao, J. , Ronald, G. , Rahme, L.G. , Stevens, E.J. , Wolfort, S.F. , Shao, J. , Tompkins, R.G. and Ausubelt, F.M. (2019) Common virulence factors for bacterial pathogenicity in plants and animals. Science, 268, 1899–1902. 10.1126/science.7604262 7604262

[mpp12876-bib-0019] Schechter, L.M. , Roberts, K.A. , Jamir, Y. , Alfano, J.R. and Collmer, A. (2004) *Pseudomonas syringae* type III secretion system targeting signals and novel effectors studied with a Cya translocation reporter. J. Bacteriol. 186, 543–555. 14702323 10.1128/JB.186.2.543-555.2004 PMC305754

[mpp12876-bib-0020] Schreiber, K.J. and Desveaux, D. (2011) AlgW regulates multiple *Pseudomonas syringae* virulence strategies. Mol. Microbiol. 80, 364–377. 21306444 10.1111/j.1365-2958.2011.07571.x

[mpp12876-bib-0021] Tang, X. , Xiao, Y. and Zhou, J.M. (2006) Regulation of the type III secretion system in phytopathogenic bacteria. Mol. Plant–Microbe Interact. 19, 1159–1166. 17073299 10.1094/MPMI-19-1159

[mpp12876-bib-0022] Valentini, M. , Gonzalez, D. , Mavridou, D.A. and Filloux, A. (2018) Lifestyle transitions and adaptive pathogenesis of *Pseudomonas aeruginosa* . Curr. Opin. Microbiol. 41, 15–20. 29166621 10.1016/j.mib.2017.11.006

[mpp12876-bib-0023] Vargas, P. , Farias, G.A. , Nogales, J. , Prada, H. , Carvajal, V. , Barón, M. , Rivilla, R. , Martín, M. , Olmedilla, A. and Gallegos, M.T. (2013) Plant flavonoids target *Pseudomonas syringae* pv. *tomato* DC3000 flagella and type III secretion system. Environ. Microbiol. Rep. 5, 841–850. 24249293 10.1111/1758-2229.12086

[mpp12876-bib-0024] Willis, D.K. , Hrabak, E.M. , Rich, J.J. , Barta, T.M. and Panopoulos, N.J. (1990) Isolation and characterization of a *Pseudomonas syringae* pv. *syringae* mutant deficient in lesion formation in bean. Mol. Plant–Microbe Interact. 3, 149–156.

[mpp12876-bib-0025] Wong, S.M. , Carroll, P.A. , Rahme, L.G. , Ausubel, F.M. and Calderwood, S.B. (1998) Modulation of expression of the ToxR regulon in *Vibrio cholerae* by a member of the two‐component family of response regulators. Infect. Immun. 66, 5854–5861. 9826365 10.1128/iai.66.12.5854-5861.1998 PMC108741

[mpp12876-bib-0026] Xin, X.F. , Kvitko, B. and He, S.Y. (2018) *Pseudomonas syringae*: what it takes to be a pathogen. Nat. Rev. Microbiol. 16, 316–328. 29479077 10.1038/nrmicro.2018.17 PMC5972017

[mpp12876-bib-0027] Yu, X. , Lund, S.P. , Greenwald, J.W. , Records, A.H. , Scott, R.A. , Nettleton, D. , Lindow, S.E. , Gross, D.C. and Beattie, G.A. (2014) Transcriptional analysis of the global regulatory networks active in *Pseudomonas syringae* during leaf colonization. mBio, 110, E425–34. 10.1128/mBio.01683-14 PMC4173789 25182327

