# Supplementary figures and images for: A revised model for the role of GacS/GacA in regulating type III secretion by Pseudomonas syringae pv. tomato DC3000
Source: Mol Plant Pathol. 2019 Oct 7;21(1):139–44. doi: 10.1111/mpp.12876 (PMC6913209; doi:10.1111/mpp.12876)

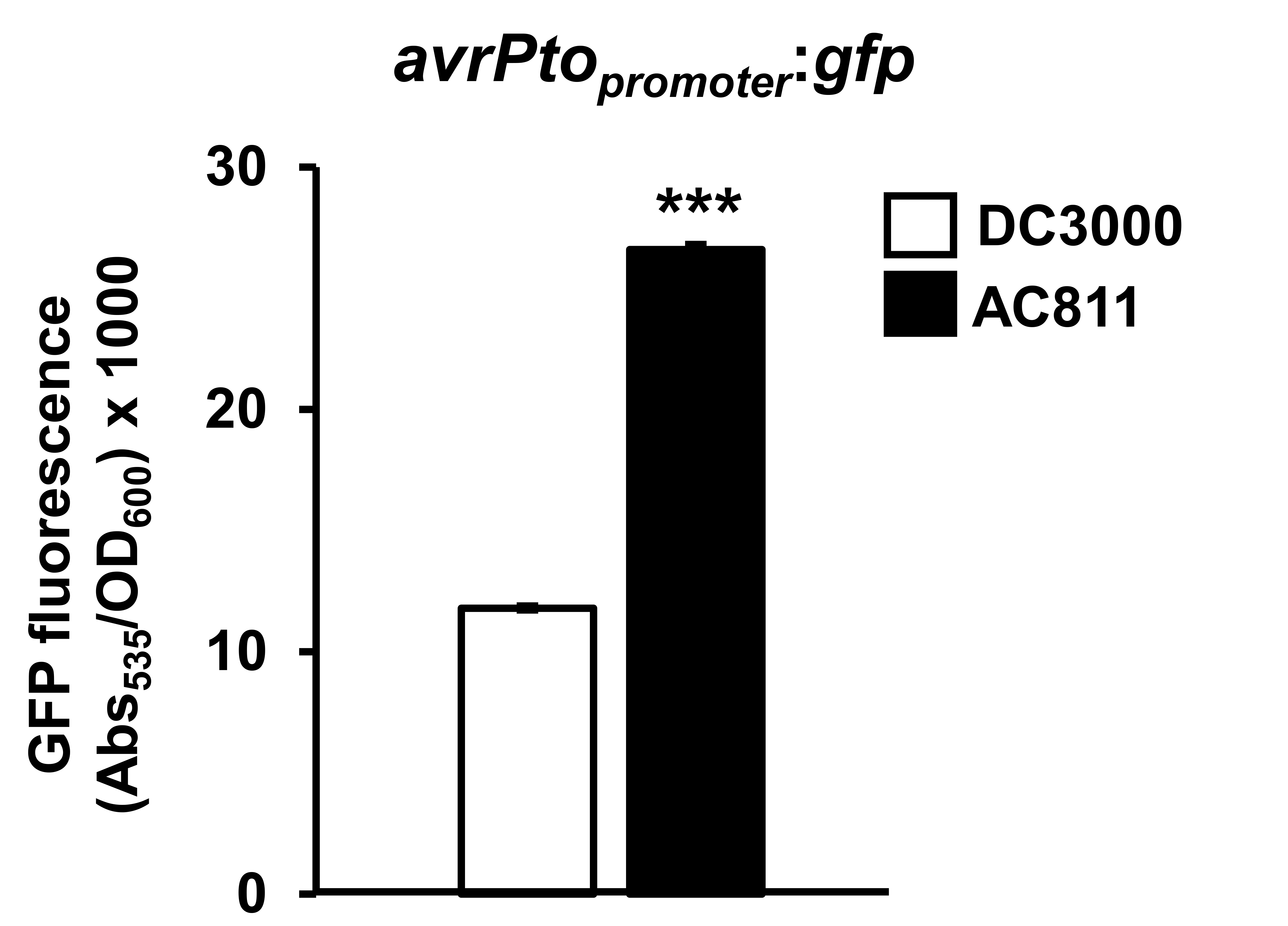

Supplement: Supplementary file 1 — Fig. S1 Hyper‐expression of avrPto occurs in AC811 cultured in KB broth prior to fructose and citric acid treatment. GFP fluorescence of DC3000 and AC811 strains carrying an avrPtopromoter:gfp reporter plasmid. Bacteria were first cultured overnight in KB broth then incubated in a minimal medium (MM) with 10 mM fructose and 400 µM citric acid. Graphed are means ± SE of GFP fluorescence at 6 h post‐inoculation normalized to OD600 and fluorescence from pProbe‐GT empty vector strains, n = 4. Asterisks denote significant difference based on t‐test, P < 0.001. Data are representative of three independent experiments. [file MPP-21-139-s001.tif]

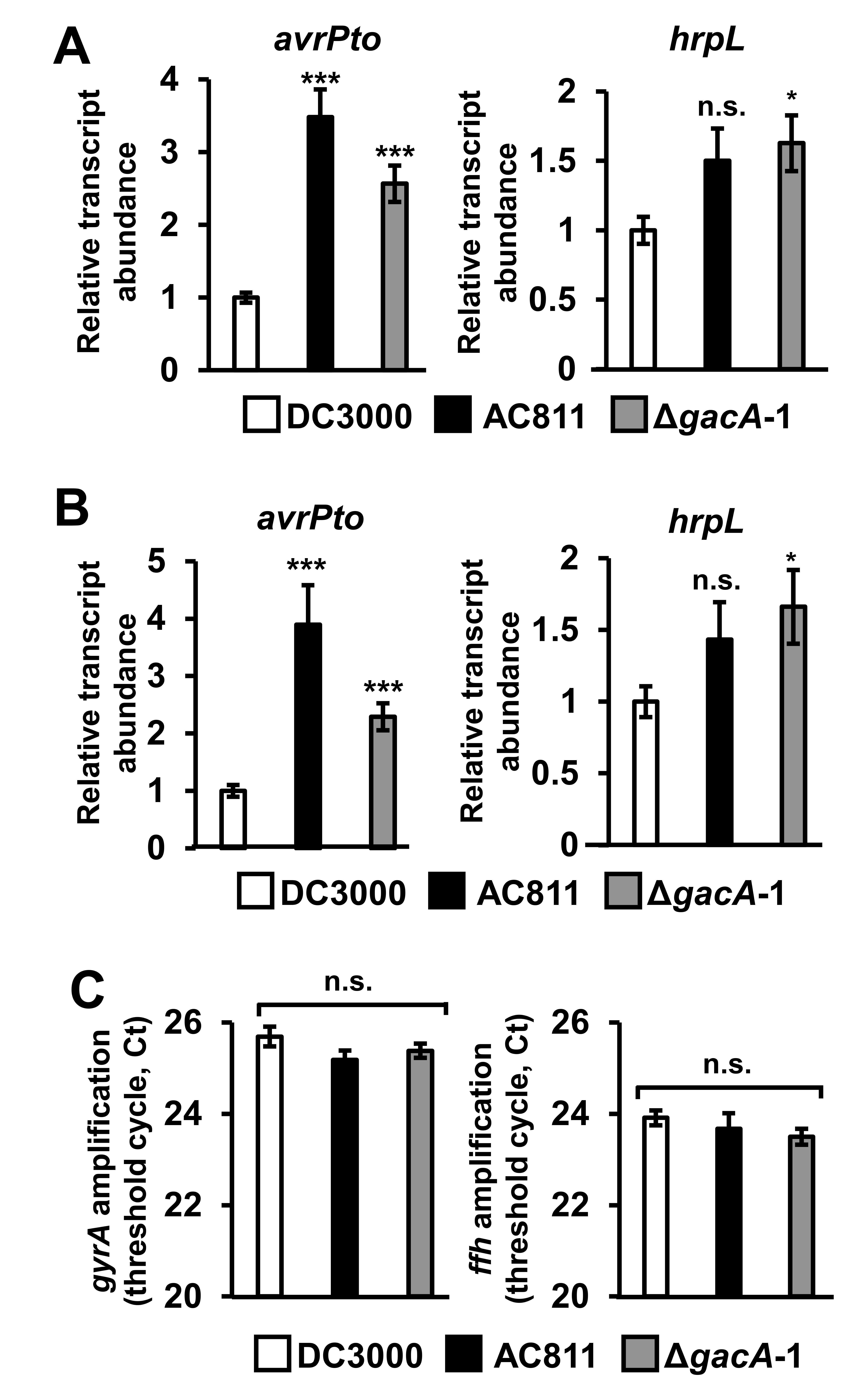

Supplement: Supplementary file 2 — Fig. S2 GacA negatively regulates the abundance of mRNA transcripts from T3SS‐associated genes. DC3000, AC811 and ΔgacA‐1 were incubated in minimal medium (MM) with 10 mM fructose and 400 µM citric acid for 2 h. The abundance of avrPto and hrpL transcripts in treated bacteria was measured by quantitative RT‐PCR. Transcript abundance was normalized to (A) gyrA or (B) ffh reference genes, followed by normalization to transcript levels in DC3000. Graphed are means ± SE from data pooled from three independent experiments, n = 10. Asterisks denote statistical significance as determined by pairwise t‐tests between DC3000 and indicated mutant strains. *P < 0.05; ***P < 0.001; n.s., no significant difference. (C) Threshold cycle (Ct) values from quantitative RT‐PCR of reference genes gyrA (left) and ffh (right). Graphed are means ± SE from data pooled from three independent experiments, n = 10. Statistical significance was determined by pairwise t‐tests; n.s., no significant difference based on P > 0.05. [file MPP-21-139-s002.tif]

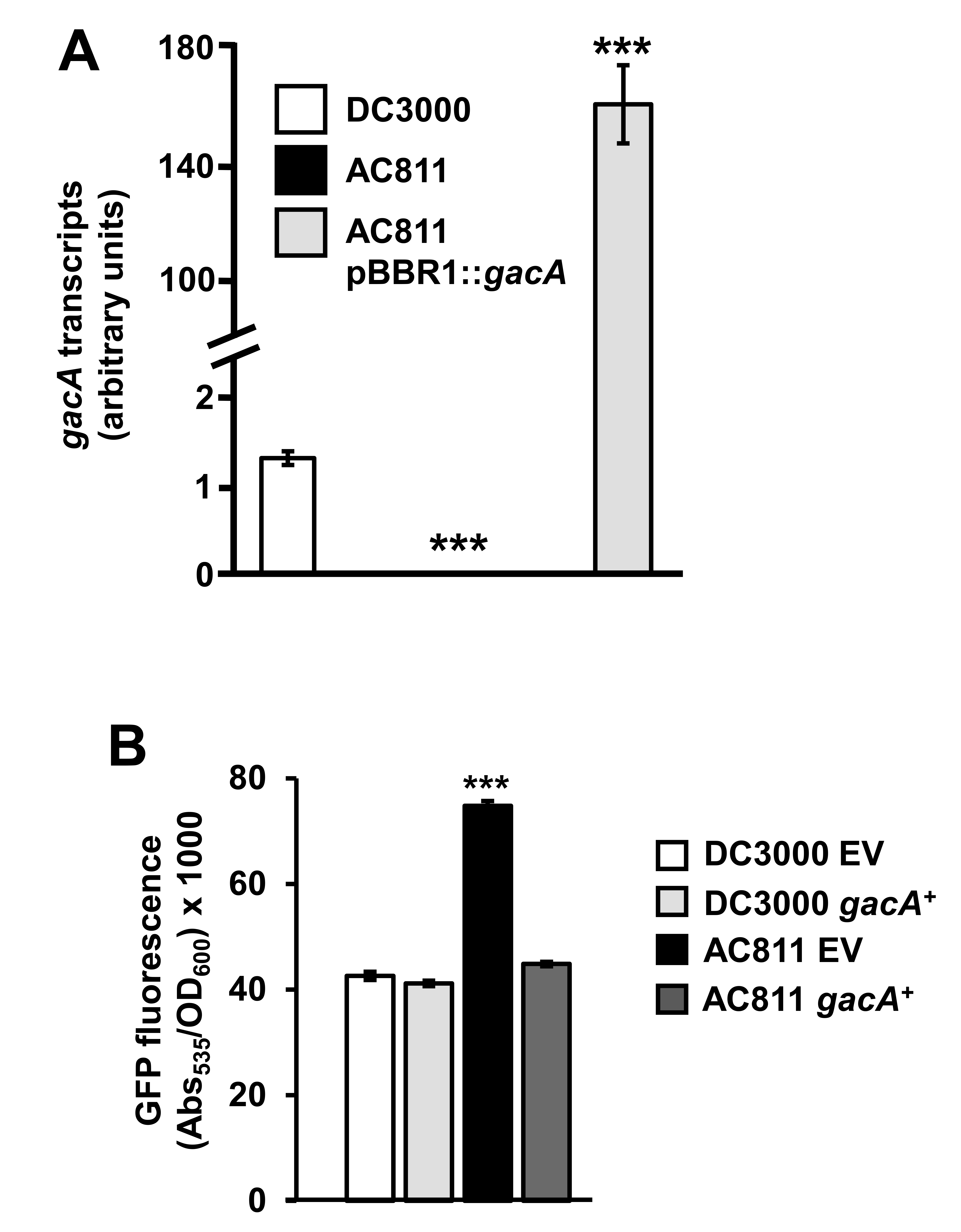

Supplement: Supplementary file 3 — Fig. S3 Expression of gacA complements the avrPto hyper‐expression phenotype of AC811. (A) Abundance of gacA transcripts measured by qRT‐PCR using gacA‐specific primers. gacA transcripts were normalized to transcripts from gyrA. Graphed are means ± SE, with data pooled from two independent experiments, n = 8. ***P < 0.001 based on two sample t‐test comparisons with DC3000. (B) GFP fluorescence of DC3000 and AC811 avrPtopromoter:gfp reporter strains carrying either a gacA‐complementing plasmid (gacA +) or empty vector (EV). Bacteria were incubated in minimal medium (MM) with 10 mM fructose and 400 µM aspartic acid. Graphed are means ± SE of GFP fluorescence at 12 h post‐inoculation normalized to OD600 and fluorescence from pProbe‐GT empty vector strains, n = 9. Data are pooled from three independent experiments. Asterisks denote significant difference between strains based on t‐test, P < 0.001. [file MPP-21-139-s003.tif]

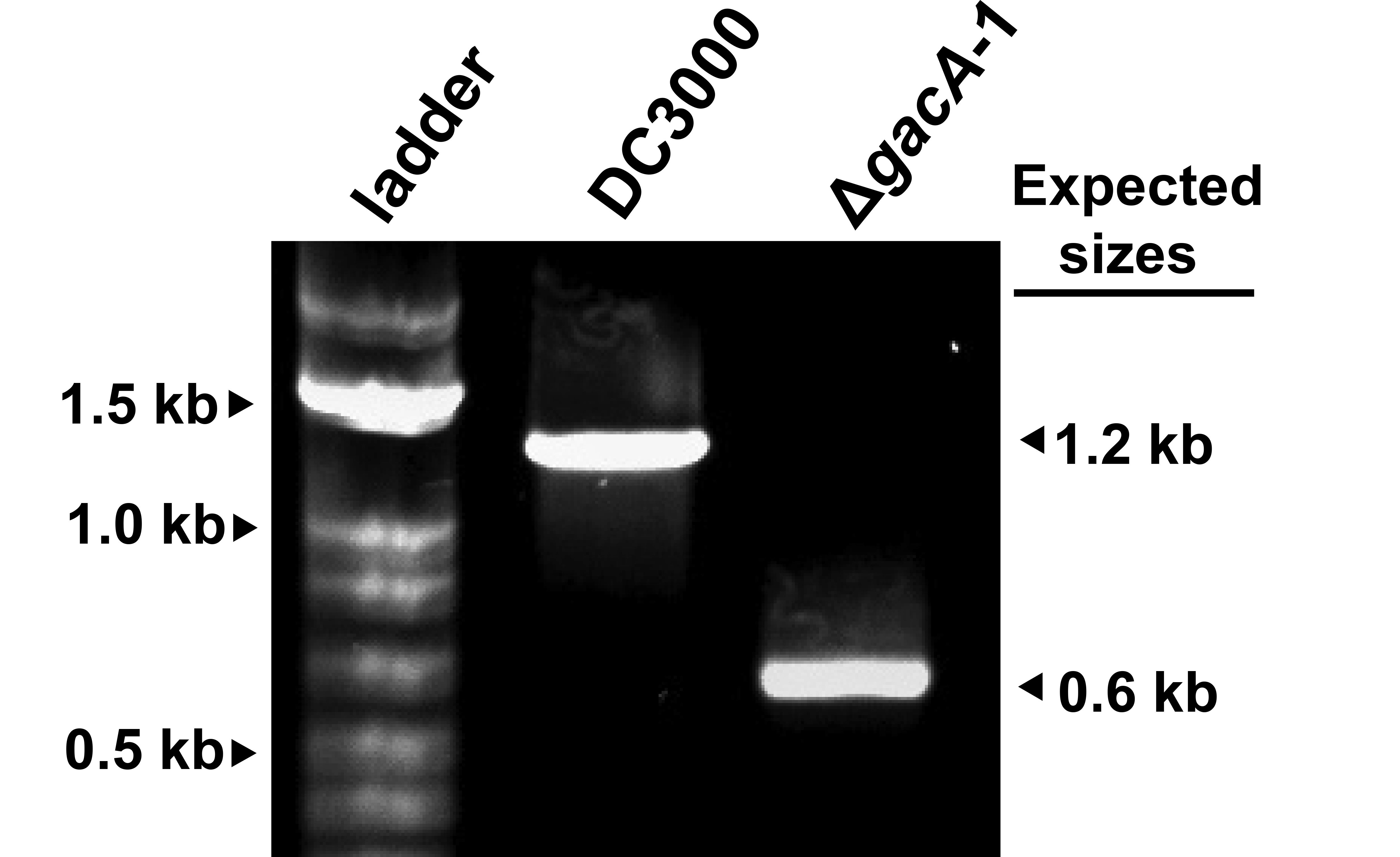

Supplement: Supplementary file 4 — Fig. S4 PCR genotyping of ΔgacA‐1 confirms deletion of gacA. A fragment of DNA containing the gacA open reading frame was PCR‐amplified from DC3000 or ΔgacA‐1 genomic DNA. Shown are PCR products separated by agarose gel electrophoresis and visualized by ethidium bromide staining. [file MPP-21-139-s004.tif]

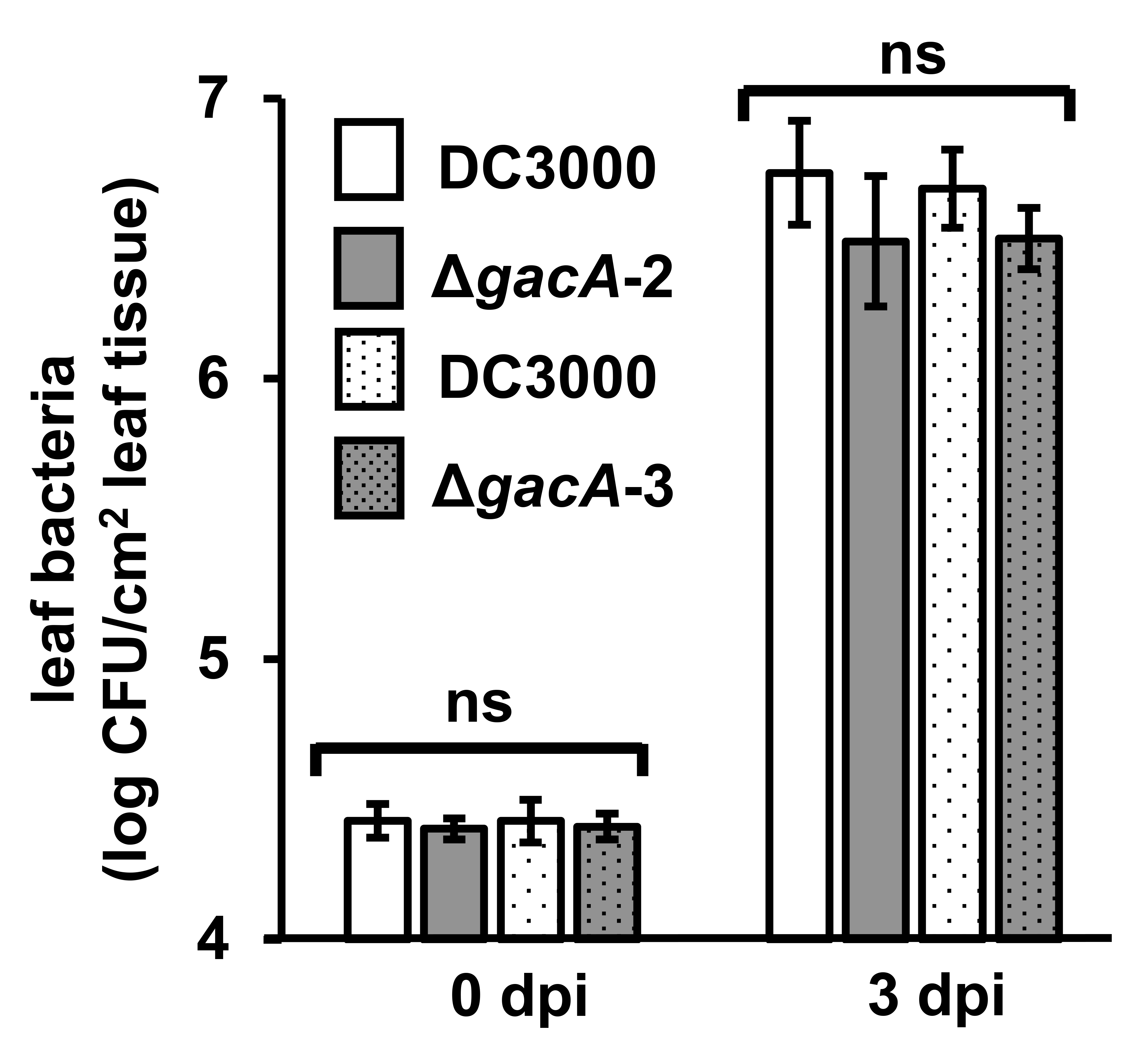

Supplement: Supplementary file 6 — Fig. S6 GacA is not required for virulence of DC3000 syringe‐infiltrated into Arabidopsis leaves. Growth of ΔgacA deletion mutants in Arabidopsis leaves infected by syringe infiltration. Solid colours indicate DC3000 obtained from G. Martin (Cornell) and its corresponding mutant ΔgacA‐2; dotted bars indicate DC3000 obtained from B. Kunkel (Wash U) and its corresponding mutant ΔgacA‐3. Graphed are means ± SE of colony‐forming units (cfus) in leaves based on serial dilution plating of leaf tissue extracts, n = 6. Data were pooled from two independent experiments. dpi, days post‐infection; ns, not significant based on ANOVA with multiple pairwise t‐test comparisons and Tukey’s post hoc HSD analysis, P < 0.05. [file MPP-21-139-s006.tif]

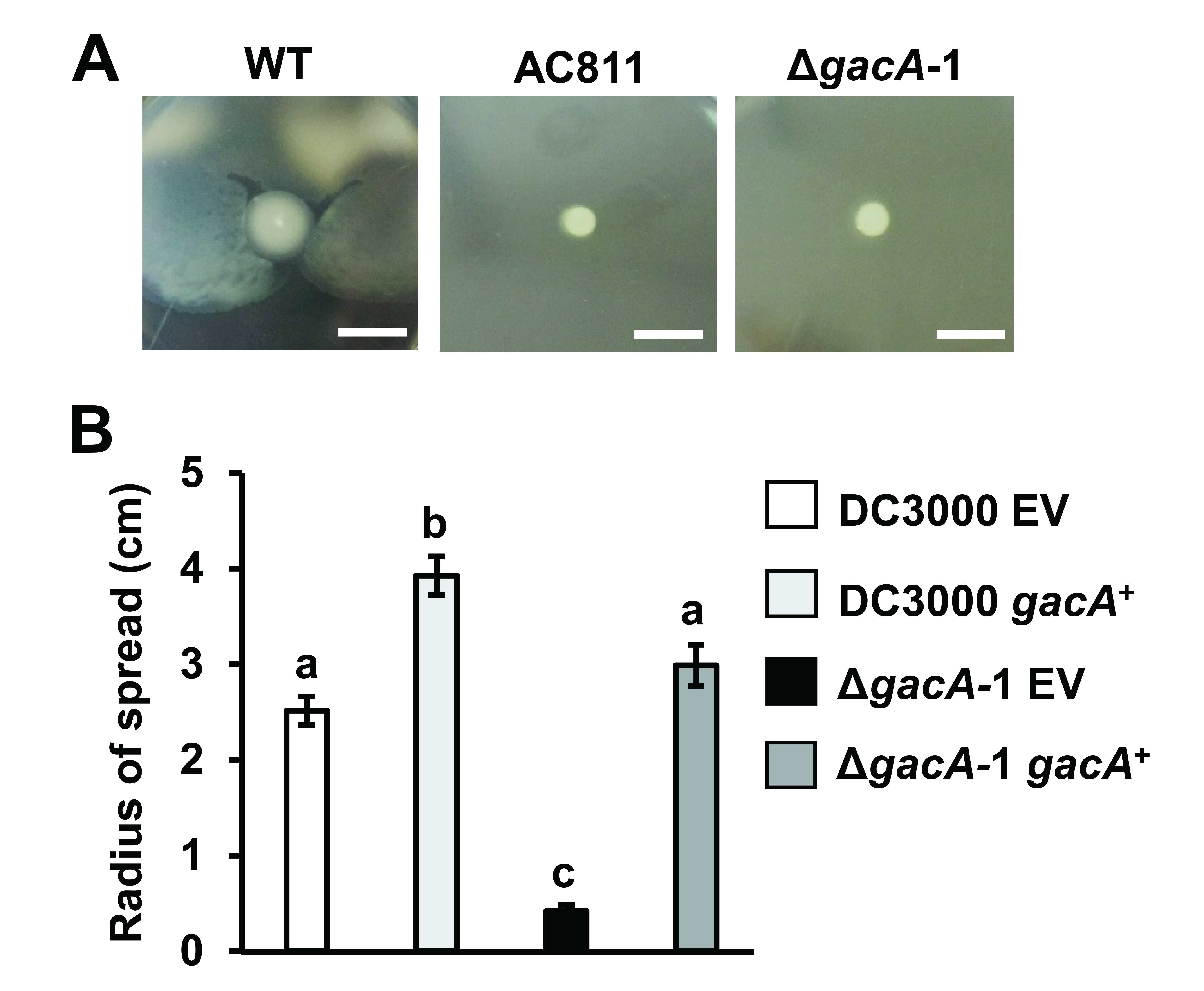

Supplement: Supplementary file 7 — Fig. S7 GacA positively regulates motility of DC3000. DC3000, AC811 and ΔgacA‐1 were individually spotted onto King's B medium (KBM) agar plates containing 0.25% agar to detect swimming motility. (A) Photographs of bacteria on swimming motility plates after 24 h. White bars show scale of 1 cm. (B) Graphed are means ± SE of radii of bacterial spread measured after 24 h on swim plates, n = 4. Data are representative of three independent experiments. [file MPP-21-139-s007.tif]
