# Supplementary material for: A revised model for the role of GacS/GacA in regulating type III secretion by Pseudomonas syringae pv. tomato DC3000
Source: Mol Plant Pathol. 2019 Oct 7;21(1):139–44. doi: 10.1111/mpp.12876 (PMC6913209; doi:10.1111/mpp.12876)
Supplement: Supplementary file 10 — Methods S1 Experimental Procedures. [file MPP-21-139-s010.docx]

**Experimental Procedures**

**Media preparation.** *P. syringae* were grown in a modified King’s B medium (KBM) (King *et al*., 1954) prepared with 1% (w/v) peptone, 1% tryptone, 0.1% MgSO_4_-7H_2_O, 0.1% K_2_HPO_4_, and 1% (v/v) glycerol. For *in vitro* T3SS-induction assays, bacteria were cultured in a modified *hrp*-inducing minimal medium (MM) [10 mM K_2_HPO_4_/KH_2_PO_4_ (pH 6.0), 7.5 mM (NH_4_)_2_SO_4_, 3.3 mM MgCl_2_, 1.7 mM NaCl]] supplemented with 0.22 µm filter-sterilized aspartic acid, citric acid and/or fructose as indicated (Huynh *et al*., 1989; Anderson *et al*., 2014). All media were autoclaved prior to use.

**Bacterial strains and culturing conditions.** *P. syringae* were stored in 20% glycerol stocks at -80°C. For all experiments, bacteria were streaked onto KBM agar plates supplemented with 50 µg/mL rifampicin and selective antibiotics as necessary (10 µg/mL tetracycline, 20 µg/mL gentamycin, 50 µg/mL chloramphenicol, 50 µg/mL spectinomycin) and grown for 2 days at room temperature prior to use.

**Measurements of *in planta* bacterial growth and AvrPto-CyaA delivery assays.** *Arabidopsis thaliana* Col-0 seeds were surface-sterilized, stratified and sown onto MS agar plates as previously described (Anderson *et al*., 2014). Two-week-old seedlings were transplanted into flats containing Sunshine mix soil (Sun Gro Horticulture, Agawam, MA, USA) and grown for 3-4 weeks in a 22°C, 10-hour day growth chamber prior to use and during infection. To prepare an inoculum, bacteria on agar plates were resuspended in 1 mL of H_2_O, then washed twice with 1 mL H_2_O using a microcentrifuge to pellet cells between washes. For syringe infiltration, suspensions of bacteria in H_2_O were adjusted to OD_600_ = 0.001 and directly infiltrated into leaves using a needleless syringe. For leaf surface inoculation, bacteria suspensions at OD_600_ = 1.0 were sprayed onto the surface of 4-week-old plants. A plastic dome was kept over surface-inoculated plants to maintain humidity. At time points indicated, a cork borer was used to punch 0.2 cm^2^ leaf disks from the infected leaf tissue. For each sample, three disks were collected from three infected leaves of a single plant and placed within a microcentrifuge tube. A plastic pestle was used to homogenize the leaf disks in 500 µL of sterile H_2_O. The resulting leaf extract was serially diluted and plated onto KB agar containing 50 µg/mL rifampicin. After 24 hours, bacteria colonies growing on the agar were counted using a stereomicroscope to aid in visualizing the colonies.

For AvrPto-CyaA delivery assays, Arabidopsis leaves were syringe-infiltrated with OD_600_ = 0.1 of DC3000 or AC811 each carrying pCPP3234, a derivative of broad-range vector pVLT35 expressing *avrPto*-*cyaA* (Schechter *et al*., 2004). Three plants were infected per strain, and three leaves were infiltrated per plant. For each sample, six 0.2 cm^2^ leaf disks were collected from a single plant, frozen in liquid nitrogen and homogenized in 600 µL 0.1 M HCl. Homogenates were centrifuged at 21,000 x *g* for 20 min at 4°C, and cAMP levels in the resulting supernatant quantified using the Direct cAMP ELISA kit (ENZO Life Sciences, Farmingdale, NY, USA) per the manufacturer’s instructions. Total sample protein from the undiluted supernatant was measured by Pierce BCA Protein Assay kit (Thermo Fisher Scientific, Waltham, MA, USA).

**Construction of *avrPto*_promoter_:*gfp* reporter and *gacA* complementation plasmids.** Primers proAvrPto-F and proAvrPto-R (Table S2) were used to PCR amplify a 120 bp fragment of the *avrPto* promoter from DC3000 genomic DNA. The PCR product was cloned upstream of a promoter-less *gfp* in pProbe-GT (Miller *et al*., 2000) previously digested with *Xba*I-*Nde*I. The resulting *avrPto*_promoter_:*gfp* reporter plasmid was introduced in *P. syringae* by tri-parental mating with an *E. coli* helper strain carrying pRK600 (Ditta *et al*., 1980). For *gacA* complementation, primers gacA-B1-F and gacA-B2-R (Table S2) were used to PCR amplify a region corresponding to the promoter and open reading frame of *gacA* from DC3000 genomic DNA. The product was amplified using primers B1 and B2 (Table S2) in a second round to add flanking *attB1* and *attB2* Gateway sequences. The resulting PCR product was Gateway cloned into entry vector pDONR207, then cloned via LR reaction into a Gateway compatible version of pBBR1MCS-1 (Kovach *et al*., 1995) kindly provided by Marc Nishimura. The *gacA* insert was amplified by PCR with M13 F/R primers, and the sequence was confirmed by Sanger sequencing.

***In vitro* analysis of T3SS gene expression.** Bacteria were scraped from the surface of an agar plate and resuspended in 1 mL of H_2_O, then washed twice in 1 mL of H_2_O using a microcentrifuge to pellet cells between washes. The washed bacteria were adjusted to OD_600_ = 1.0, then diluted 10-fold into 1 mL of MM supplemented with 10 mM fructose and 400 µM citric acid. Cultures were placed on an orbital platform shaker set to 200 rpm and incubated at 20°C under constant light. At times indicated, 50 µL samples were transferred into wells of Greiner Bio-One (Kremsmünster, Austria) black µClear 384-well microtiter plates, and GFP fluorescence in each well measured at 485_excitation_/535_emission_ wavelengths using a Tecan Spark 10M microplate reader. Fluorescence measurements were normalized to Absorbance at 600 nm (Abs_600_) readings and to Abs_600_-normalized fluorescence readings from identical cultures of control strains carrying empty pProbe-GT. Protein extraction and immunoblot analysis of AvrPto was performed as described previously (Anderson *et al*., 2014).

For measurements of *avrPto* expression in DC3000 cultured first in KB broth, bacteria from two day old KB agar plates were inoculated into 4 mL KB liquid broth with 50 µg/mL rifampicin. Cultures were grown overnight at 28°C with 200 RPM shaking to an OD_600_ ~ 1.0. One mL of culture was transferred to a micro-centrifuge tube and cells washed three times with 1 mL of H_2_O. Suspension of bacteria in H_2_O were normalized to 1 mL OD_600_ = 0.5, and inoculated into MM with 10 mM fructose and 400 µM citric acid at a final OD_600_ = 0.05. Bacterial cultures were incubated at 28°C for 6 hr with continuous shaking, and GFP fluorescence was quantified by Tecan Spark 10M microplate reader as described previously.

**Generation of Δ*gacA* deletions in DC3000.** The upstream and downstream fragments of *gacA* were PCR-amplified with primer pairs Pto_3024mt-1/Pto_3024mt-2 and Pto_3024mt-3/Pto_3024mt-4, respectively, and cloned into pK18mobsacB suicide vector (Schäfer *et al*., 1994). The resulting construct was transformed into DC3000 by tri-parental mating, and kanamycin-resistant merodiploid colonies cultured for 1-3 days in KBM with 50 µg/mL rifampicin prior to plating on KBM with 15% sucrose for *sacB* counter-selection. Sucrose-resistant colonies were plated on KBM with or without 30 µg/mL kanamycin, and screened by PCR with primers gacA-F and gacA-R to identify deletion of *gacA*.

**Quantitative RT-PCR analysis of bacterial gene expression.** Bacterial strains were grown on agar plates for two days, washed three times with 1 mL water, and adjusted to OD_600_ = 2.0. Bacteria were then inoculated into *hrp*-inducing minimal medium to a final OD_600_ = 0.02. Cells were incubated for 2-3 hr at 20°C under continuous light with 200 RPM shaking. Cultures were then transferred to microcentrifuge tubes, and bacterial cells were pelleted by centrifugation and immediately flash frozen in liquid nitrogen. Samples were stored at -80°C prior to use. RNA extraction using Trizol reagent (Thermo Scientific) and cDNA synthesis using random hexamer primers were performed as described previously (Anderson *et al*., 2014). qRT-PCR was performed with 10 µL reactions containing 5 µL of SsoAdvanced Universal SYBR Green Supermix (Bio-Rad, Hercules, CA, USA), 3 µL of a solution containing 0.5 µM each transcript-specific primer, and 2 µL of cDNA template. qRT-PCR was performed using a Bio-Rad C1000 Thermal Cycler with CFX96 Real-Time System. The abundance of *gyrA* transcripts was measured in each sample as a reference for normalization, along with an alternate reference gene, *ffh*. Transcript abundance was calculated by the formula Transcript Abundance = PCR efficiency^-(Ct[^*^gene^*^]-Ct[^*^gyrA^*^])^ as described (Pfaffl, 2001). PCR efficiency (PE) of each reaction was calculated using LinRegPCR (Ramakers *et al*., 2003). Sequences of primers used for qRT-PCR are provided in Table S2.

**Motility assays.** KBM agar plates were prepared with 0.5% or 0.25% agar for swarming and swimming assays, respectively (Kinscherf and Willis, 1999). *P. syringae* were resuspended in 1 mL of H_2_O, then washed twice in 1 mL of H_2_O using a microcentrifuge to pellet cells between washes. The washed bacteria were resuspended to OD_600_ = 1.0 in H_2_O, and 5 µL of the bacterial suspension was spotted in the center of the plate. Plates were stored inverted in the dark at room temperature for 48 hours before imaging or measuring the radius of bacterial spread.

**References**

**Anderson J.C., Wan Y., Kim Y-M., Pasa-Tolic L., Metz T.O., and Peck S.C.** (2014) Decreased abundance of type III secretion system-inducing signals in Arabidopsis *mkp1* enhances resistance against *Pseudomonas syringae*. Proc Natl Acad Sci USA **111**, 6846–6851.

**Ditta G., Stanfield S., Corbin D., Helinski D.R., and Donald Helinskl C.R.** (1980) Broad host range DNA cloning system for Gram-negative bacteria: Construction of a gene bank of *Rhizobium meliloti*. Genetics **77**, 7347–735.

**Huynh T.V., Dahlbeck D., and Staskawicz B.J.** (1989) Bacterial blight of soybean: regulation of a pathogen gene determining host cultivar specificity. Science **245**, 1374–1377.

**King E.O., Ward M.K., and Raney D.E.** (1954) Two simple media for the demonstration of pyocyanin and fluorescein. J Lab Clin Med **44**, 301–307.

**Kinscherf T.G. and Willis D.K.** (1999) Swarming by *Pseudomonas syringae* B728a requires *gacS* (*lemA*) and *gacA* but not the acyl-homoserine lactone biosynthetic gene *ahlI*. J Bacteriol **181**, 4133–4136.

**Kovach M.E., Elzer P.H., Hill D.S., Robertson G.T., Farris M.A., Roop R.M., and Peterson K.M.** (1995) Four new derivatives of the broad-host-range cloning vector pBBR1MCS, carrying different antibiotic-resistance cassettes. Gene **166**, 175–176.

**Miller W.G., Leveau J.H.J., and Lindow S.E.** (2000) Improved *gfp* and *inaZ* broad-host-range promoter-probe vectors. Mol Plant Microbe Interact **13**, 1243-1250.

**Pfaffl M.W.** (2001) A new mathematical model for relative quantification in real-time RT-PCR. Nucleic Acids Res **29**, e45.

**Ramakers C., Ruijter J.M., Lekanne Deprez R.H., and Moorman A.F.M.** (2003) Assumption-free analysis of quantitative real-time polymerase chain reaction (PCR) data. Neurosci Lett **339**, 62–66.

**Schäfer A., Tauch A., Jäger W., Kalinowski J., Thierbach G., and Pühler A.** (1994) Small mobilizable multi-purpose cloning vectors derived from the *Escherichia coli* plasmids pK18 and pK19: selection of defined deletions in the chromosome of *Corynebacterium glutamicum*. Gene **145**, 69–73.

**Schechter L.M., Roberts K.A., Jamir Y., Alfano J.R., and Collmer A.** (2004) *Pseudomonas syringae* type III secretion system targeting signals and novel effectors studied with a Cya translocation reporter. J Bacteriol **186**, 543–555.
